# Guided block matching and 4-D transform domain filter projection denoising method for dynamic PET image reconstruction

**DOI:** 10.1186/s40658-023-00580-5

**Published:** 2023-09-25

**Authors:** Lin Xin, Weihai Zhuo, Haikuan Liu, Tianwu Xie

**Affiliations:** Institute of Radiation Medicine, Fudan University, 2094 Xietu Road, Shanghai, 200032 China

**Keywords:** Block matching and 4-D transform domain filter, Dynamic PET, Projection denoising

## Abstract

**Purpose:**

Dynamic PET is an essential tool in oncology due to its ability to visualize and quantify radiotracer uptake, which has the potential to improve imaging quality. However, image noise caused by a low photon count in dynamic PET is more significant than in static PET. This study aims to develop a novel denoising method, namely the Guided Block Matching and 4-D Transform Domain Filter (GBM4D) projection, to enhance dynamic PET image reconstruction.

**Methods:**

The sinogram was first transformed using the Anscombe method, then denoised using a combination of hard thresholding and Wiener filtering. Each denoising step involved guided block matching and grouping, collaborative filtering, and weighted averaging. The guided block matching was performed on accumulated PET sinograms to prevent mismatching due to low photon counts. The performance of the proposed denoising method (GBM4D) was compared to other methods such as wavelet, total variation, non-local means, and BM3D using computer simulations on the Shepp–Logan and digital brain phantoms. The denoising methods were also applied to real patient data for evaluation.

**Results:**

In all phantom studies, GBM4D outperformed other denoising methods in all time frames based on the structural similarity and peak signal-to-noise ratio. Moreover, GBM4D yielded the lowest root mean square error in the time-activity curve of all tissues and produced the highest image quality when applied to real patient data.

**Conclusion:**

GBM4D demonstrates excellent denoising and edge-preserving capabilities, as validated through qualitative and quantitative assessments of both temporal and spatial denoising performance.

## Introduction

Positron emission tomography (PET) is a non-invasive imaging technique that collects information on the annihilation of positrons and electrons via high-energy photon detection. When used in conjunction with 2-deoxy-2-[18F]fluoro-D-glucose (FDG), PET is widely recognized as a valuable tool for detecting pathological changes in various applications, including neurology, cardiology, and oncology [1]. Dynamic PET is particularly valuable in oncology due to its ability to visualize and quantify radiotracer uptake [2]. Additionally, dynamic PET enables analysis by the time activity curve (TAC) based on the compartment model [3]. However, compared to static PET, dynamic PET images suffer from higher noise levels in single voxels, which poses a significant challenge to denoising techniques [2]. Noise can be reduced by reconstruction methods, such as the iterative-based ordered subset expectation maximization (OSEM) reconstruction method, but excessive blurring may occur with an increase in the number of subsets and iteration times [4]. Furthermore, the PET denoising algorithm helps to obtain images at the same noise level with reduced injection activity and scanning time. Thus, the development of denoising algorithms suitable for PET imaging is an active area of research. Common denoising techniques such as Gaussian, median, and Wiener filters are not very effective in reducing noise in PET images due to spillover activity or low efficiency [5]. To address this, various edge-preserving or non-local algorithms have been proposed to denoise the PET images, mostly post-reconstruction, including bilateral filtering, wavelet-based techniques, guided image filtering, temporal and spatiotemporal smoothing techniques, and non-local filters like non-local mean (NLM) and block-matching 3D (BM3D) along with its higher dimensional form BM4D and BM5D [6–17]. Combined with anatomical information, the performance of various filters can be further improved [18–21].

BM3D is conducted by block matching followed by 3-D transform domain filtering. The block-matching process generates grouped fragments by collecting similar patches and stacking them into a 3-D group. BM3D exploits the similarity between the blocks to enhance the sparsity so that the transformed coefficients of grouped fragments can be better shrunk. The similarity between small blocks at the different spatial and temporal positions in PET images is common, which motivates the use of grouping and collaborative filtering for PET images, especially dynamic PET images [9, 10]. Ote et al. [8] proposed a post-reconstruction kinetics-induced BM5D filtering to denoise dynamic PET images. Radioactive decay is well-modeled as a Poisson process. Block matching and transform-domain collaborative filtering based on Anscombe transformation has been proven successful in denoising images with Poisson noise [22]. However, the reconstructed is decidedly non-Poisson [23].

Given the unique characteristics of dynamic PET images, simplifying the block-matching process using an accumulative activity map, as outlined in this work, can be highly beneficial. This involves performing block matching in 2D on an accumulative sinogram (referred to as guide image in this work), allowing corresponding 3D dynamic sinogram blocks to be easily grouped and stacked into 4D stacks, which reduces the likelihood of mismatching and minimizes computational costs associated with grouping. By stacking similar patches and filtering in the 4-D transform domain of the dynamic PET sinogram, the sparsity of transformed coefficients of the patches can be further reduced based on kinetic information in sinograms, enabling TAC denoising and spatial denoising to be conducted simultaneously.

Here, we developed guided block matching and 4-D transform domain filter (GBM4D) projection denoising method for dynamic PET image reconstruction. To evaluate the performance of the GBM4D algorithm, a simulation sinogram of two digital phantoms and a clinical head PET sinogram was included in this work for quantitative and qualitative evaluation.

## Methods

### Algorithm

#### Overview of GBM4D

**Fig. 1:**
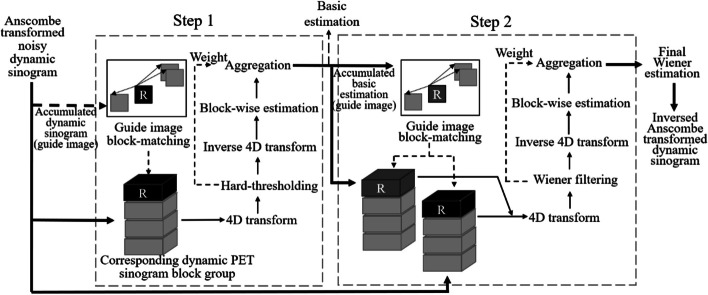
Flowchart of guided block matching and 4-D transform domain filter projection denoising method for dynamic PET image reconstruction. Steps 1 and 2 are repeatedly conducted for each block

The noise of dynamic PET sinogram can be well-modeled as Poisson distribution. BM3D and BM4D methods are designed for Gaussian noise. Thus, the generalization Anscombe transform was first applied to the sinogram. The general procedure of GBM4D is demonstrated in Fig. 1.

The final estimation is obtained by inversed Anscombe transformation of GBM4D filtered sinogram. The algorithm consists of two steps: hard thresholding step and Wiener filtering step. Each of the processes consists of block-matching and collaborative filtering by shrinkage in a 4-D transform domain as follows:

- Find blocks that are similar to the reference block in the cumulative PET sinogram. 2-D blocks at the corresponding spatial position in each scanning frame are stacked together to generate 3-D sinogram blocks. All similar blocks were stacked together to form a 4-D array (group).
- Perform collaborative filtering of the group, then aggregate the sinograms by returning the filtered 3-D blocks to the original position.

Detailed of the Anscombe transformation and denoising Step 1 and Step 2 in Fig. 1 will be described in the following sections.

#### Guided block matching and grouping in Step 1

Considering Poisson noise in noisy sinogram $z:X, T \to {\mathbb{R}}$ as the form

1 $$\begin{array}{*{20}c} {z\left( {x, t} \right) = P\left( {y\left( {x,t} \right)} \right), \quad x\varepsilon X,t\varepsilon T} \\ \end{array}$$

where ${\mathcal{P}}$ is independent random Poisson distribution, and y is the true sinogram. The Anscombe transformation was first conducted on the sinogram before Step 1. In this case, only pure Poisson noise is considered. After denoising process (both Step 1 and Step 2 in Fig. 1), inverse Anscombe exact unbiased transformation were conducted using validated database to avoid biased inverse result in low-count Poisson image [22].

After general Anscombe transformation, it is reasonable to assume that the noisy sinogram $z:X, T \to {\mathbb{R}}$ as the form

2 $$\begin{array}{*{20}c} {z\left( {x, t} \right) = y\left( {x,t} \right) + \eta \left( {x,t} \right), x\varepsilon X,t\varepsilon T} \\ \end{array}$$

where $x$ is the 2-D spatial position of the sinogram $X \subset {\mathbb{Z}}^{2}$, t is the temporal position of dynamic sinograms $T \subset {\mathbb{R}}^{ + }$ and $\eta \left( \cdot \right)\sim N\left( {0,\sigma^{2} } \right)$. In the block-matching process, the similarity of two blocks were measured by the inverse of the $\ell^{2}$-distance. If the true image y were available, the block distance would be measured as:

3 $$\begin{array}{*{20}c} {d\left( {Z_{{x_{R} }}^{t} , Z_{x}^{t} } \right) = \frac{{\left\| {Y_{{x_{R} }}^{t} - Y_{x}^{t} } \right\|_{2}^{2} }}{{N^{2} }}} \\ \end{array}$$

where $\left\| \cdot \right\|_{2}$ denotes the $\ell^{2}$-norm, and the blocks $Z_{{x_{R} }}^{t} \;{\text{and}}\;Z_{x}^{t}$ are in $z$ and are located at $x_{R}$ and $x \in X$ at time $t \in T$ and $x_{R}$ are located at the reference position, and blocks $Y_{{x_{R} }}^{t} { }\;{\text{and}}\;{ }Y_{x}^{t}$ are located at $x_{R}$ and $x \in X$ at time $t \in T$ in $y$, N denotes the block size. In realistic situations, only noisy blocks $Z_{{x_{R} }}^{t} { }\;{\text{and}}\; Z_{x}^{t}$ are available. Therefore, the distance is estimated as:

4 $$\begin{array}{*{20}c} {\hat{d}\left( {Z_{{x_{R} }}^{t} , Z_{x}^{t} } \right) = \frac{{\left\| {Z_{{x_{R} }}^{t} - Z_{x}^{t} } \right\|_{2}^{2} }}{{N^{2} }}} \\ \end{array}$$

The distance is a non-central chi-square random variable with expectation

5 $$\begin{array}{*{20}c} {E\left\{ {\hat{d}\left( {Z_{{x_{R} }}^{t} , Z_{x}^{t} } \right)} \right\} = d\left( {Z_{{x_{R} }}^{t} , Z_{x}^{t} } \right) + 2\sigma^{2} } \\ \end{array}$$

and variance

6 $$\begin{array}{*{20}c} {{\text{var}} \left\{ {\hat{d}\left( {Z_{{x_{R} }}^{t} ,Z_{x}^{t} } \right)} \right\} = \frac{{8\sigma^{4} }}{{N^{2} }} + \frac{{8\sigma^{4} d\left( {Z_{{x_{R} }}^{t} , Z_{x}^{t} } \right)}}{{N^{2} }}} \\ \end{array}$$

The variance grows asymptotically with ${\mathcal{O}}\left( {\sigma^{4} } \right)$. For dynamic PET sinograms, the noise in each frame is relatively large compared with accumulative sinograms due to fewer photon counts in each frame. For larger $\sigma$, the probability densities of different $\hat{d}\left( {Z_{{x_{R} }}^{t} ,{ }Z_{x}^{t} } \right)$ might overlap heavily. Such mismatches can worsen the sparsity in the 4-D groups, which may lead to inefficiency in the collaborative filtering process. Previous work used coarse prefiltering to avoid such mismatch [8, 9], which is realized by linear transform on blocks and hard-thresholding. In this work, coarse prefiltering was applied to avoid mismatch along with the introduction of guide image (accumulated transformed PET sinograms). The distance after coarse prefiltering can be written as:

7 $$\begin{array}{*{20}c} {\hat{d}\left( {Z_{{x_{R} }} , Z_{x} } \right) = \frac{{\left\| {\Upsilon^{\prime } \left( {{\mathcal{T}}\left( {Z_{{x_{R} }}^{{{\text{guide}}}} } \right)} \right) - \Upsilon^{\prime } \left( {{\mathcal{T}}\left( {Z_{x}^{{{\text{guide}}}} } \right)} \right)} \right\|_{2}^{2} }}{{N^{2} }}} \\ \end{array}$$

where $\Upsilon^{\prime }$ is the hard-thresholding operator and ${\mathcal{T}}$ is the linear transformation and

8 $$\begin{array}{*{20}c} {Z_{{x_{R} }}^{{{\text{guide}}}} = \mathop \sum \limits_{t} Z_{{x_{R} }}^{t} \;{\text{and }}\;Z_{x}^{{{\text{guide}}}} = \mathop \sum \limits_{t} Z_{x}^{t} } \\ \end{array}$$

As stated previously, the transformed accumulated PET sinogram has smaller noise compared with each dynamic PET sinogram frame. Therefore, we used accumulated transformed PET sinograms as guide images. For Step 1, the blocking set at $x_{R}$, $S_{{x_{R} }}$, generated by block matching contains the blocks in each frame at $x_{R}$ and $x$ where $Z_{{x_{R} }}$ and $Z_{x}$ of guide image is similar:

9 $$\begin{array}{*{20}c} {S_{{x_{R} }} = \left\{ {x\varepsilon X:\hat{d}\left( {Z_{{x_{R} }}^{{{\text{guide}}}} , Z_{x}^{{{\text{guide}}}} } \right) \le \tau_{{{\text{match}}}} } \right\}} \\ \end{array}$$

where $\tau_{{{\text{match}}}}$ is the maximum $\hat{d}\left( {Z_{{x_{R} }} , Z_{x} } \right)$ for which the block is considered similar to reference block. The block group is formed base on $S_{{x_{R} }}$ by stacking $Z_{{xS_{{x_{R} }} }}^{t}$ into a 4-D array. The array is of size $N \times N \times \left| T \right| \times \left| {S_{{x_{R} }} } \right|$.

#### Collaborative filtering using hard-thresholding in Step 1

The collaborative filtering of $Z_{{x\varepsilon S_{{x_{R} }} }}^{t}$ is conducted in 4-D domain using hard-thresholding in Step 1 in Fig. 1. This filtering can maintain good sparsity while obtaining the information of the correlation 1) between the pixels of a single block 2) between the pixels at the corresponding spatial position in grouped blocks 3) between the pixels at the corresponding temporal position in grouped blocks.

**Fig. 2:**
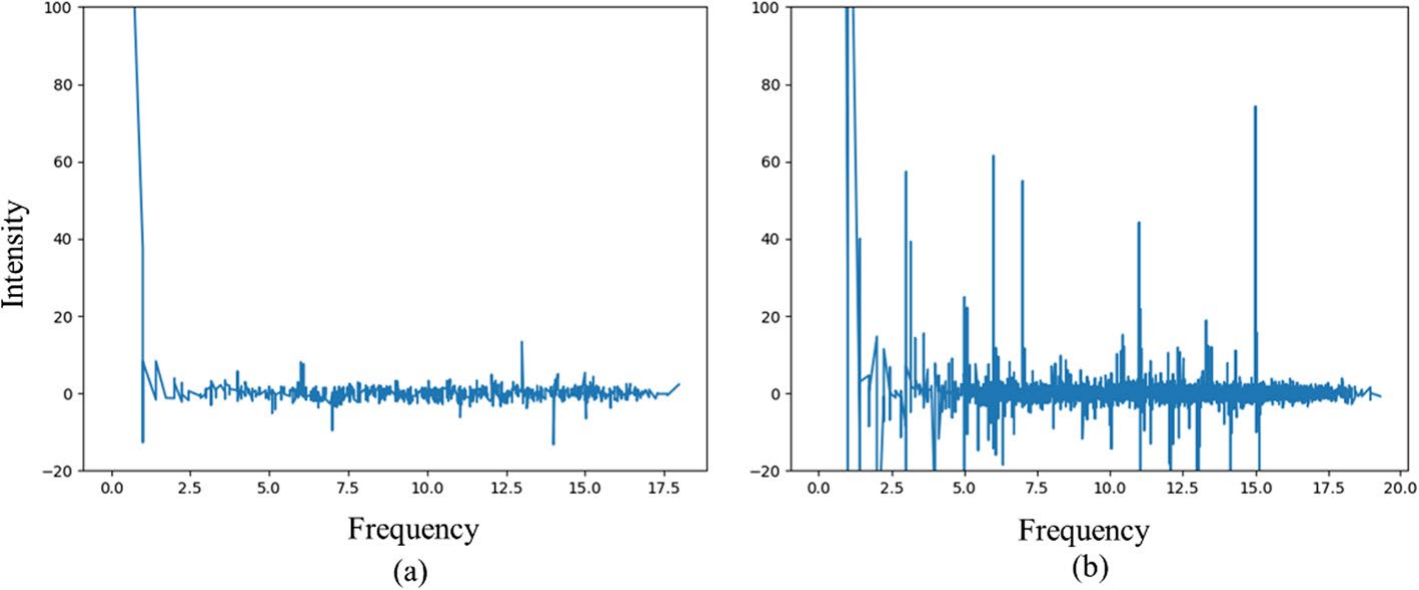
Example of **a** 3-D spectrum of a group in BM3D method performed on a single sinogram frame of dynamic PET transformed by 3-D linear transform **b** 4-D spectrum of the group in GBM4D method performed on a dynamic PET sinogram transformed by 4-D linear transform. The 4-D spectrum is sparser than the 3-D spectrum

Similar 3-D patches were stacked to form 4-D patches to conduct collaborative filtering. For BM3D, denoising takes advantage of the sparsity in the spectrum of 3-D similar block groups. As demonstrated in Fig. 2, the sparsity of the 3-D block spectrum is enhanced by introducing kinetic information since the temporal correlation in the signals is also considered in GBM4D. The hard-thresholding filtering in the 4-D domain is expressed as:

10 $$\begin{array}{*{20}c} {\hat{\user2{Y}}_{{x\varepsilon S_{{x_{R} }} }}^{t} = {\mathcal{T}}_{4D}^{ - 1} \left( {\Upsilon \left( {{\mathcal{T}}_{4D} \left( {{\varvec{Z}}_{{x\varepsilon S_{{x_{R} }} }}^{t} } \right)} \right)} \right)} \\ \end{array}$$

${\mathcal{T}}_{4D}$ and ${\mathcal{T}}_{4D}^{ - 1}$ are the normalized 4-D linear transform and inverse transform. In this work, 3-D DCT in spatial and temporal domain followed by 1D DCT transform in group direction and its inverse transform are applied. ${\Upsilon }$ denotes the hard-thresholding process in Step 1 in Fig. 1:

11 $$\Upsilon \left( \upsilon \right) = \left\{ {\begin{array}{*{20}c} {0,} & {\left| \upsilon \right| \le \lambda_{4D} \sigma } \\ {\upsilon ,} & {{\text{otherwise}}} \\ \end{array} } \right.$$

Here, $\lambda_{4D}$ is set to 2.8 based on a previous study [24]. After aggregation by weighted average (detailed stated in 2.1.5), the filtered blocks were returned to the original position to form the basic estimation of sinogram $\hat{y}^{{{\text{basic}}}} \left( {x, t} \right)$ in Step 1 in Fig. 1.

#### Grouping and collaborative wiener filtering in Step 2

Step 1 gives a basic estimation of true dynamic PET sinogram $\hat{y}^{{{\text{basic}}}} \left( {x, t} \right)$. By accumulated sinogram based on $\hat{y}^{{{\text{basic}}}} \left( {x, t} \right)$, the guide image in Step 2 is calculated as:

12 $$\begin{array}{*{20}c} {\hat{y}^{{{\text{guide}}}} \left( x \right) = \mathop \sum \limits_{t} \hat{y}^{{{\text{basic}}}} \left( {x, t} \right)} \\ \end{array}$$

The denoising is further improved by performing the grouping in Step 2 in Fig. 1 using the basic estimation and applying Wiener filtering.

As stated previously, the accumulated basic estimation, referring to guide image, is significantly attenuated, which helps to find more accurate block groups. The match blocks in Step 2 were generated as:

13 $$\begin{array}{*{20}c} {S_{{x_{R} }}^{{{\text{Wie}}}} = \left\{ {x\varepsilon X:\frac{{\left\| {\hat{Y}_{{x_{R} }}^{{{\text{guide}}}} - \hat{Y}} \right\|_{2}^{2} }}{{N^{2} }} \le \tau_{{{\text{match}}}}^{{{\text{wiener}}}} } \right\}} \\ \end{array}$$

$\hat{Y}_{{S_{{x_{R} }}^{{{\text{Wie}}}} }}^{{{\text{basic}}_{{\text{t}}} }}$ as the stacked block of grouped basic estimation blocks and $Z_{{S_{{x_{R} }}^{{{\text{Wie}}}} }}^{{{\text{basic}}_{{\text{t}}} }}$ as the stacked block of grouped noisy sinogram blocks. The Wiener shrinkage coefficient is calculated as:

14 $$\begin{array}{*{20}c} {\hat{\user2{Y}}_{{S_{{x_{R} }}^{{{\text{Wie}}}} }}^{t} = {\mathcal{T}}_{4D}^{ - 1} \left( {W_{{S_{{x_{R} }}^{{{\text{Wie}}}} }} {\mathcal{T}}_{4D} \left( {{\varvec{Z}}_{{S_{{x_{R} }}^{{{\text{Wie}}}} }}^{t} } \right)} \right)} \\ \end{array}$$

where

15 $$\begin{array}{*{20}c} {W_{{S_{{x_{R} }}^{{{\text{Wie}}}} }} = \frac{{\left| {{\mathcal{T}}_{4D} \left( {\hat{Y}_{{S_{{x_{R} }}^{{{\text{Wie}}}} }}^{{{\text{basic}}_{t} }} } \right)} \right|^{2} }}{{\left| {{\mathcal{T}}_{4D} \left( {\hat{Y}_{{S_{{x_{R} }}^{{{\text{Wie}}}} }}^{{{\text{basic}}_{t} }} } \right)} \right|^{2} + \sigma^{2} }}} \\ \end{array}$$

By using the Wiener filtering, power spectrum of the basic estimate can be used to filter the groups by minimizing the least-square of the difference between modeled and filtered signals. After aggregation by weighted average, the Weiner filtered blocks were returned to the original position to form the final estimation of sinogram.

#### Aggregation by weighted average in Step 1 and Step 2

By returning the filtered block to the original position, the estimation of $\hat{y}^{{{\text{basic}}}} \; {\text{and }}\; \hat{y}^{{{\text{wiener}}}}$ can be calculated for both Step 1 and Step 2 in Fig. 1, which is called aggregation. Weighted average aggregation was adopted in this work as:

16 $$\begin{array}{*{20}c} {\hat{y}\left( {x, t} \right) = \frac{{\mathop \sum \nolimits_{{x_{R} \epsilon X}} \mathop \sum \nolimits_{{x_{m} \epsilon S_{{x_{R} }} }} \omega_{{x_{R} }} \hat{Y}_{{x_{m} }}^{{x_{R} }} \left( {x, t} \right)}}{{\mathop \sum \nolimits_{{x_{R} \epsilon X}} \mathop \sum \nolimits_{{x_{m} \epsilon S_{{x_{R} }} }} \omega_{{x_{R} }} \chi_{{x_{m} }} \left( {x, t} \right)}}, \quad \forall x\varepsilon X} \\ \end{array}$$

where $\chi_{{x_{m} }} :X \to \left\{ {0, 1} \right\}$ is the characteristic function of the block and $\omega_{{x_{R} }}$ is the weight function based on [7]. Kaiser window is also part of the weights to reduce border effects [7, 25].

### Experimental setup

#### Computer simulation

We performed computer simulation on the Shepp–Logan phantom (SLP) [26] and a digital brain phantom developed by Martin A. Belzunce et al. [27] For SLP, only physical decay was considered when generating the sinogram. The reconstruction image size was 8 × 128 × 128 × 128. For the digital brain phantom, TACs of gray matter, white matter and tumor tissue were calculated by compartment model. The pharmacokinetic parameters of gray matter, white matter and tumor were $K_{1}$ = 0.1104, 0.0622, 0.0640 mL/min/mL, $k_{2}$ = 0.1910, 0.1248, 0.0890 mL/min/mL, $k_{3}$ = 0.1024, 0.0070, 0.0738 mL/min/mL. $F_{{\text{v}}}$ were set to 0. The input function was extracted from previous work [28]. According to a previous study [2], a dynamic PET of 8 $\times$ 6 min was performed. The tumor size is 4 × 4 × 4 pixels. The size of sinograms is 8 × 128 × 128 × 128. The sinogram is generated by forward radon transformation using Python scikit-image toolkit. After generating the noise-free sinogram, Poisson noise was added to the sinogram assuming the total photon count of 5 $\times$ 10^8^ according to previous simulation work [8]. The dynamic PET was then reconstructed using 2D-OSEM with twenty iterations and eight subsets with matrix size of 128 × 128 and no post-filter. The update equation for the OSEM can be briefly described as:

$$\hat{f}_{j}^{{\left( {n,b} \right)}} = \frac{{\hat{f}_{j}^{{\left( {n,b - 1} \right)}} }}{{\mathop \sum \nolimits_{{i^{\prime } \epsilon S_{b} }} H_{{i^{\prime } j}} }}\mathop \sum \limits_{{i\epsilon S_{b} }} H_{ij} \frac{{p_{i} }}{{\mathop \sum \nolimits_{k} H_{ik} \hat{f}_{j}^{{\left( {n,b - 1} \right)}} }}$$

where $f$ is the image under reconstruction, j and k are voxel indices, n is iteration number b is the subset number, $i$ is the sinogram indices and $S_{b}$ is subset $b$. $p$ is the sinogram voxel measurement, and $H$ is the system matrix generated by inversed radon transform using Python scikit-image toolkit. The size of the reconstruction image was 8 × 128 × 128 × 128, and the voxel size is 1.5 × 1.5 × 1.5 mm^3^. All simulation and reconstruction were performed based on PYTHON.

To validate the performance of GBM4D compared with other algorithms, total variation, wavelet, non-local means (NLM), and BM3D method were applied to denoise the sinogram using skimage toolkit in PYTHON except for BM3D. Total variation denoising aims at obtaining an image that has a minimal total variation norm. The weight of the total variation is set to 0.1 [29]. The non-local means algorithm replaces the value of a pixel by an average of a selection of other similar non-local pixels values. The patch size and the search area of NLM are set to 5 × 5 and 13 × 13 pixels [30]. Wavelet denoising uses the wavelet representation of the image to removed noise by shrinking all coefficients toward zero by a given amount. Soft thresholding and Bayes shrinking methods were adopted for wavelet denoising [31]. During the denoising process, the robust wavelet-based estimator of the noise standard deviation was applied based on a previous study [32]. Before the denoising, generalized Anscombe transformation was performed on all sinograms since all the methods were designed based on Gaussian noise instead of Poisson noise. The exact unbiased inverse of the Anscombe transformation was then performed on the denoised sinogram before the reconstruction. To exclude the effect of the reconstruction algorithm, the ground truth images were the reconstruction of the noiseless sinogram.

For the quantitative evaluation of different denoising methods, the structural similarity (SSIM) and peak signal-to-noise ratio (PSNR) were calculated. SSIM measures the similarity of ground truth and denoised image based on the degradation of structural information [33]:

17 $$\begin{array}{*{20}c} {{\text{SSIM}} = \frac{{\left( {2\mu_{g} \mu_{d} + c_{1} } \right)\left( {2\sigma_{gd} + c_{2} } \right)}}{{\left( {\mu_{g}^{2} + \mu_{d}^{2} + c_{1} } \right)\left( {\sigma_{g}^{2} + \sigma_{d}^{2} + c_{2} } \right)}}} \\ \end{array}$$

where $\mu_{g}$, $\mu_{d}$ are the mean of the ground truth image and the denoised image, $\sigma_{g}^{2}$, $\sigma_{d}^{2}$ are the deviation of the ground truth image and the denoised image, $\sigma_{gd}$ is the covariance of the ground truth image and the denoised image, $c_{1}$ = $c_{2}$ = 0.01^2^. PSNR was calculated in this work to measure the image quality at the pixel level:

18 $$\begin{array}{*{20}c} {{\text{PSNR}} = 20\log_{10} \frac{{{\text{peak}}}}{{{\text{RMSE}}}}\;{\text{dB}}} \\ \end{array}$$

where RMSE is the root mean square error between the ground truth image and the denoised image and peak is the peak value of the ground truth image. SSIM and PSNR in each time frame were measured. The TACs of different tissues were measured in the volume of interest of 4 × 4 × 4 pixels. The region of interest (ROI) positions can be seen in Fig. 7. To quantitatively measure the temporal smoothing performance of GBM4D, RMSE of TACs in different tissues measured from denoised images compared with the ground truth were calculated.

#### Real patient data

The real patient data in this retrospective study are based on an open accessed dynamic PET list-mode sinogram data source, which is acquired on a Siemens Biograph mMR, using amyloid tracer 18F-florbetapir, provided by Avid Radiopharmaceuticals, Inc., a wholly-owned subsidiary of Lilly [34, 35]. The data extraction and reconstruction of the dynamic PET data were performed offline using NiftyPET. The reconstruction was performed using histogram mode with image matrix sizes of 344 × 344 and no post-filter. [36]. The frame setting is also 8 $\times$ 6 min. The dynamic PET images were then reconstructed using OSEM with four iterations and eight subsets. The reconstruction PET image size was 8 × 127 × 344 × 344. The direct sinogram and oblique sinograms were denoised separately. The denoising methods and parameters are the same as stated in the previous section.

## Result

### Computer simulation

**Fig. 3:**
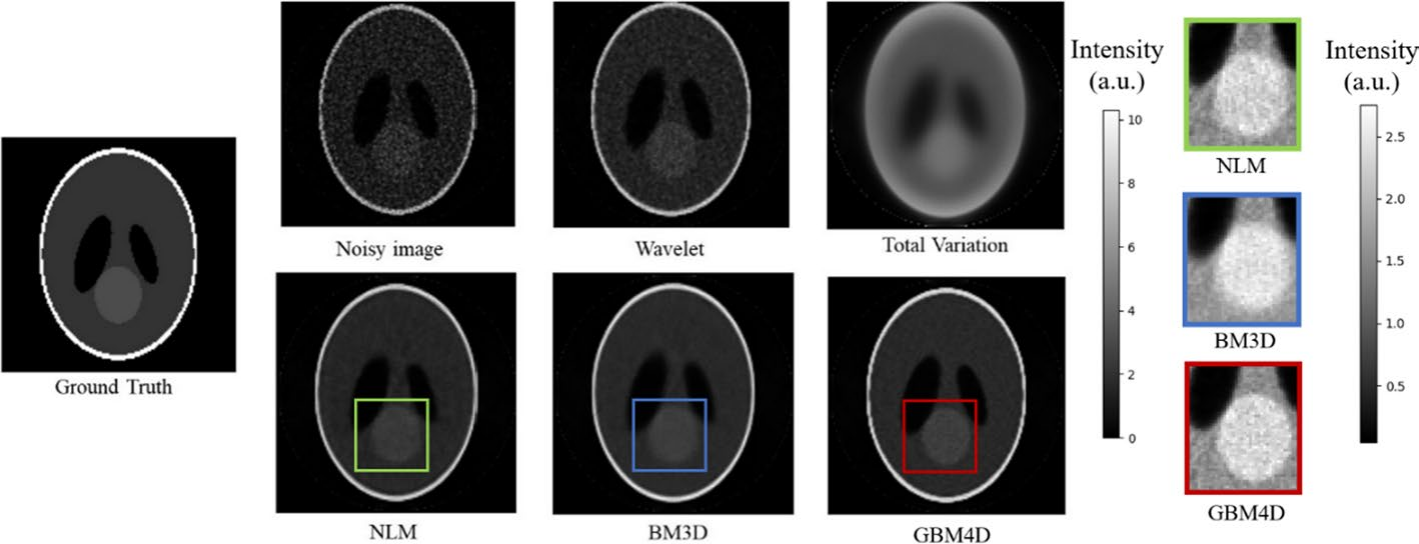
A slice of the SLP for the 8th frame and its reconstructed image before and after applying different the various denoising approaches

**Fig. 4:**
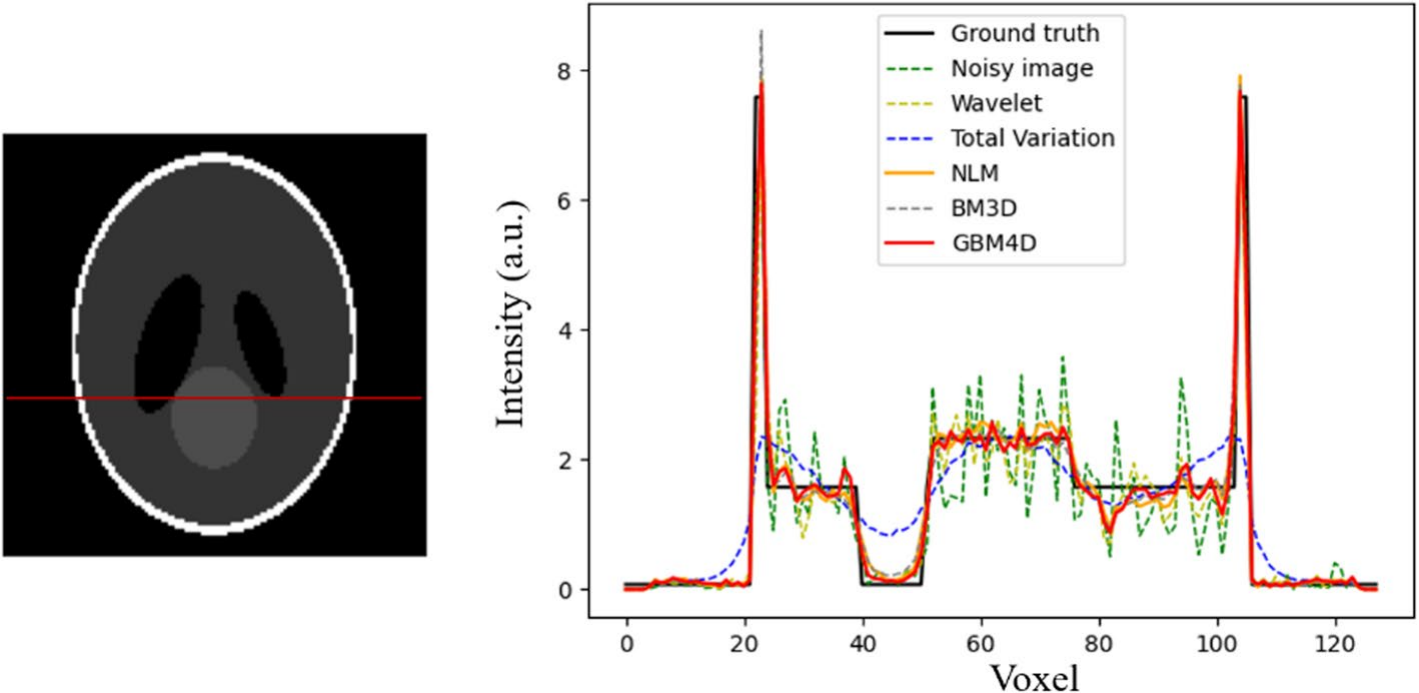
A horizontal profile of the SLP for the 8th frame and its reconstructed image before and after applying different denoising approaches

To qualitatively validate the algorithm when only considering physical attenuation, Fig. 3 shows the 8^th^ frame of the reconstructed dynamic PET image of SLP before and after applying different denoising approaches with 1.4M counts per slice. The wavelet method can preserve the structural details, but the noise was not properly removed. The total variation method tends to over-smooth the image, causing the loss of image details. An obvious image distortion can be seen in NLM denoised image. GBM4D shows better performance in edge-preserving compared with BM3D. As shown in Fig. 4, the denoising performance of GBM4D can be further demonstrated with the horizontal profile of the reconstruction image before denoising and after different denoising approaches. GBM4D denoised image shows great consistency with the ground truth. To quantitatively measure the performance of different denoising approaches, SSIM and PSNR were measured as shown in Table 1. For both indexes, GBM4D shows the best denoising performance compared with other approaches. SLP is a phantom with rather uniform tissue distribution locally, causing less chance of mismatching in BM3D and better accuracy of denoised image patch in NLM. Therefore, the performances between different algorithms are similar except for total variation denoising. The respectively small noise and artifact in noisy images also contribute to the overall better denoising performance of different algorithms. The differences of indexes in different frames caused by the photon counts were also small for all algorithms.

**Table 1:** SSIM and PSNR between the ground truth and reconstructed SLP image after applying different denoising approaches for different frames

	Frame	1	2	3	4	5	6	7	8
SSIM	Total variation	0.64	0.64	0.64	0.64	0.64	0.64	0.65	0.65
	Wavelet	0.96	0.96	0.96	0.96	0.96	0.95	0.95	0.95
	NLM	0.97	0.97	0.97	0.97	0.97	0.97	0.97	0.97
	BM3D	0.96	0.96	0.96	0.96	0.96	0.96	0.96	0.96
	GBM4D	0.98	0.98	0.98	0.98	0.98	0.98	0.98	0.98
PSNR (dB)	Total variation	17.65	17.66	17.68	17.69	17.70	17.72	17.74	17.76
	Wavelet	25.56	25.46	25.40	25.29	25.24	25.15	25.05	24.97
	NLM	27.28	27.24	27.24	27.20	27.17	27.17	27.10	27.10
	BM3D	26.24	26.22	26.20	26.16	26.14	26.13	26.08	26.04
	GBM4D	28.68	28.75	28.80	28.84	28.87	28.90	28.90	28.89

**Fig. 5:**
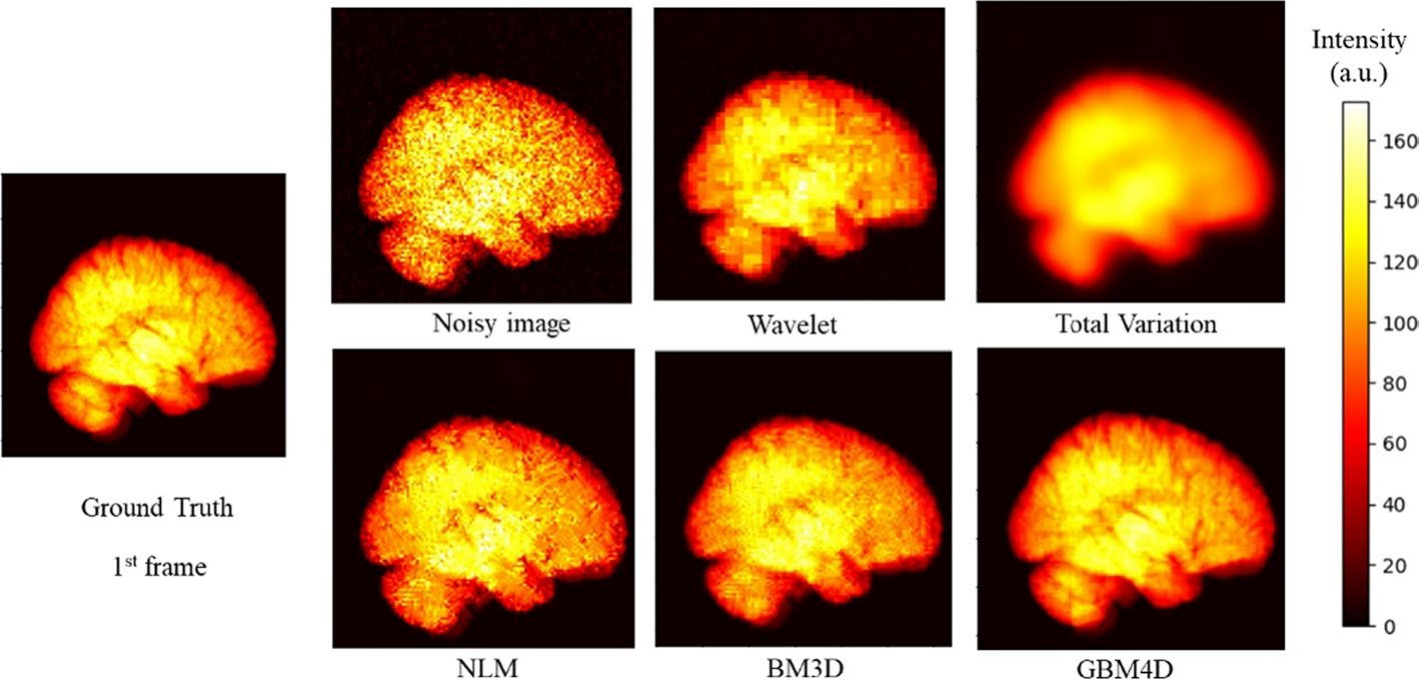
A slice of digital brain phantom sinogram (projection) for the 1^st^ frame before and after applying different denoising approaches

**Fig. 6:**
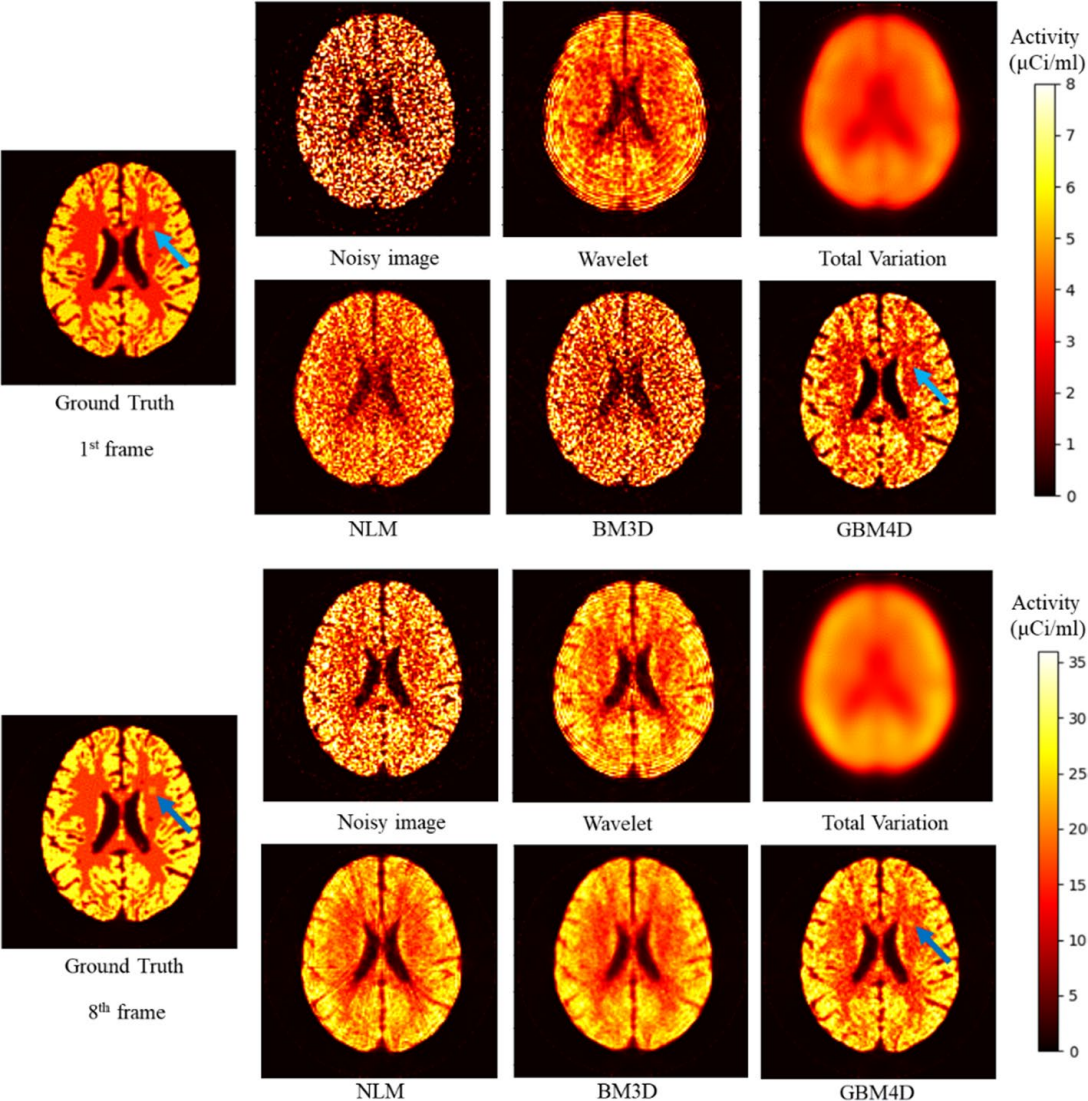
A slice digital brain phantom for the 1st and 8th frame and its reconstructed image before and after applying different denoising approaches (the arrow points out the tumor)

**Fig. 7:**
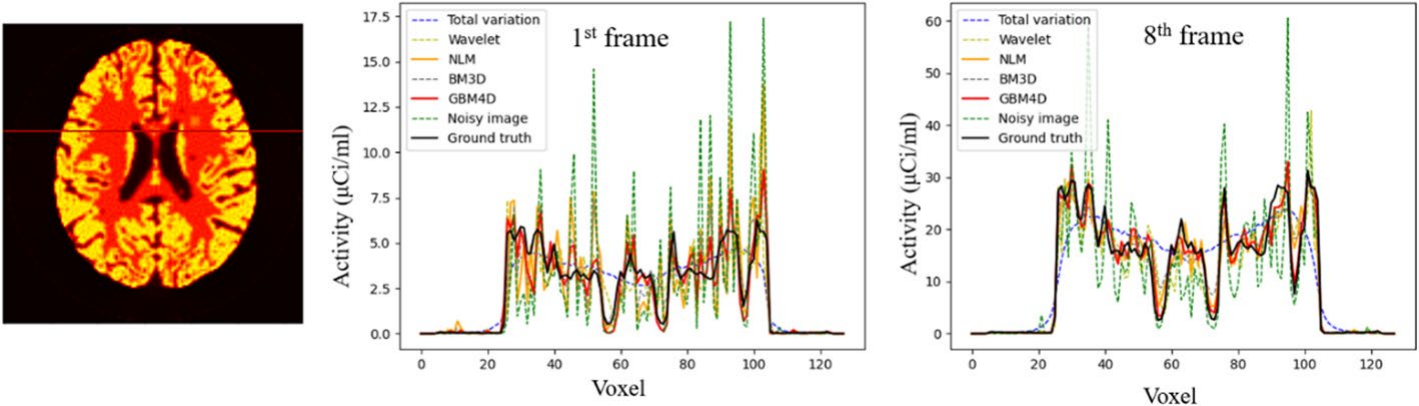
A horizontal profile of the digital brain phantom for the 1st and the 8th frame before and after applying different denoising approaches

Figure 5 shows the digital brain phantom sinogram before and after applying different denoising approaches. When considering biokinetic in the phantom, GBM4D still showed better denoising performance in sinogram perspective. Figure 6 shows the digital brain phantom images reconstructed from the sinogram before and after applying different denoising approaches. When considering the simulated digital brain phantom, GBM4D showed better denoising performance, especially for the 1^st^ frame which is of fewer photon counts. It is shown in Fig. 6 that tumor tissue can be only detected when using the GBM4D denoising technique. Because wavelet is not able to represent discontinuities along edges or curves in images or objects efficiently, wavelet denoised reconstructed image showed ring artifacts caused by the streaking artifacts in wavelet denoised sinogram. Similar to the previous result, the total variation method tends to over-smooth the image. Compared with NLM and BM3D, GBM4D showed greater denoising and edge-preserving performance, especially for the frame of lower photon counts. Such a result can be also demonstrated in Fig. 7. Figure 7 shows the great consistency of the ground truth and the GBM4D denoised image horizontal profile. To quantitatively evaluate the performance of GBM4D and other denoising approaches, SSIM and PSNR were calculated. Table 2 shows GBM4D significantly improved SSIM and PSNR for each frame (p < 0.001). The differences of indexes in different frames caused by the photon counts were also reduced while using GBM4D compared with other denoising methods. For wavelet, NLM and BM3D methods, the denoising performance for frames with fewer photon counts was significantly inferior to frames with higher counts.

**Table 2:** SSIM and PSNR between the ground truth and digital brain phantom image after applying different denoising approaches for different frames

	Frame	1	2	3	4	5	6	7	8
SSIM	Total variation	0.89	0.88	0.88	0.88	0.88	0.87	0.87	0.87
	Wavelet	0.88	0.92	0.92	0.93	0.93	0.94	0.94	0.94
	NLM	0.76	0.93	0.95	0.95	0.96	0.96	0.96	0.96
	BM3D	0.87	0.95	0.96	0.96	0.96	0.96	0.96	0.96
	GBM4D	0.94	0.97	0.98	0.98	0.98	0.98	0.98	0.98
PSNR (dB)	Total variation	17.93	17.61	17.49	17.44	17.40	17.34	17.32	17.30
	Wavelet	16.88	18.42	18.64	19.07	19.39	19.62	19.80	19.77
	NLM	12.95	19.44	20.77	21.14	21.57	21.83	21.86	22.06
	BM3D	16.23	20.74	21.36	21.65	21.89	21.91	22.05	22.08
	GBM4D	19.24	23.13	24.21	24.60	24.85	24.92	24.95	25.02

**Fig. 8:**
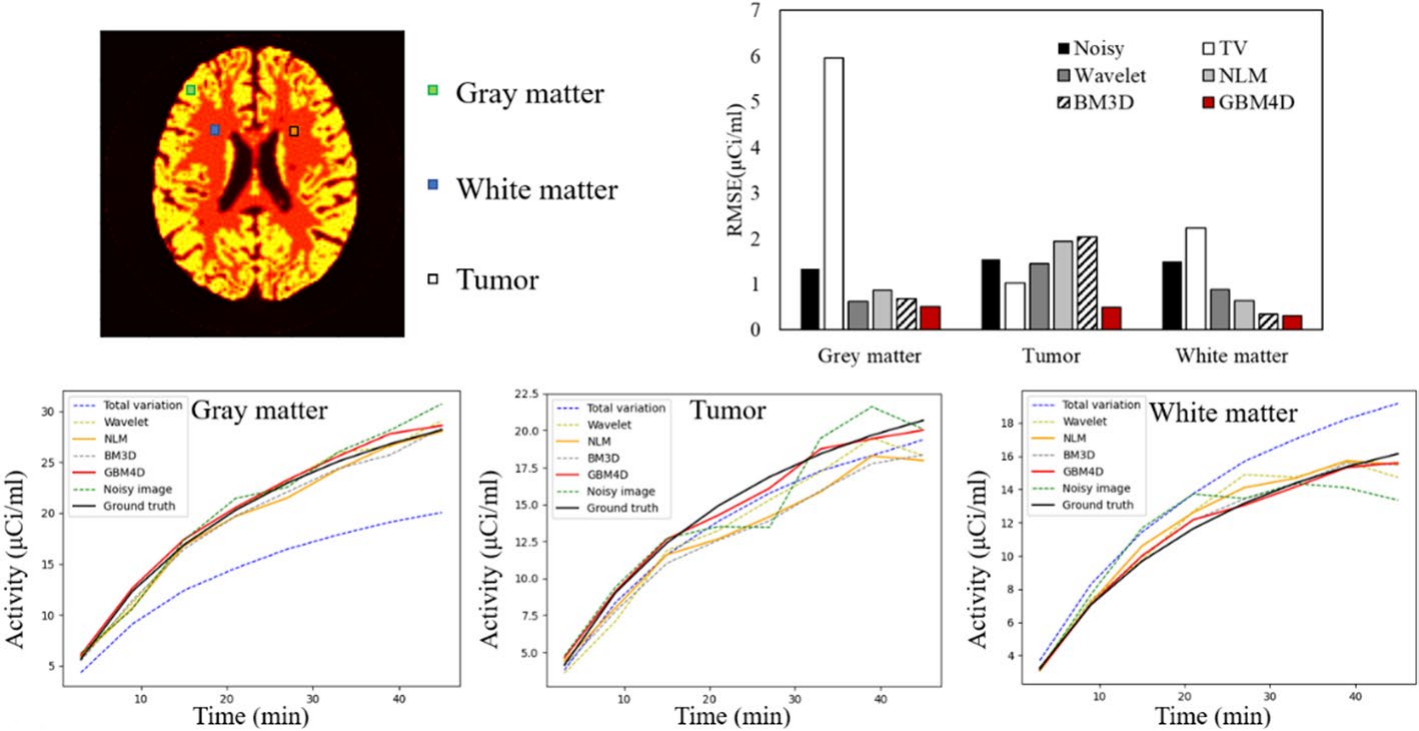
The TACs of different tissues of the digital brain phantom before and after applying different the various denoising approaches along with the RMSE of TACs when applied different denoising approaches

Figure 8 shows that GBM4D also has the best temporal denoising performance compared with other denoising approaches considered in this work. The GBM4D denoised image shows great consistency with ground truth in TACs of different tissue. The RMSE of different tissue TACs when using GBM4D is the lowest among all (0.51, 0.49, 0.31 for gray matter, tumor and white matter).

### Real patient data

**Fig. 9:**
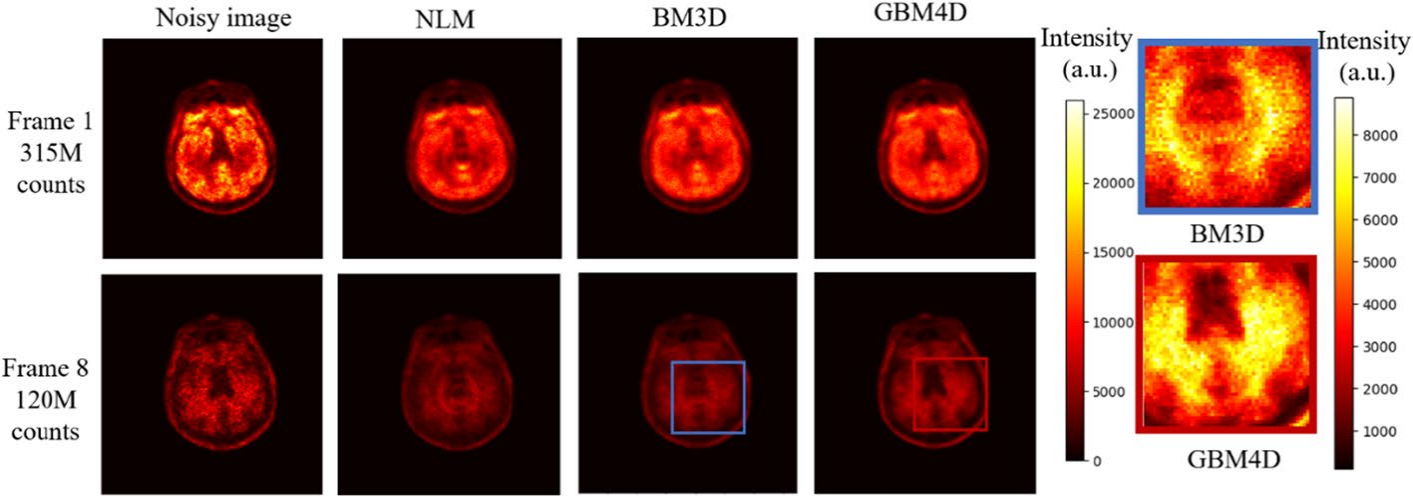
A slice of patient brain PET image before and after applying different denoising approaches

**Fig. 10:**
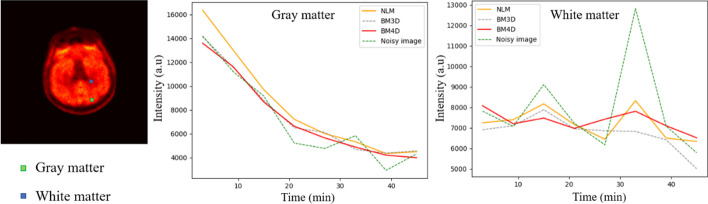
The TACs of different tissues of the real patient dynamic PET before and after applying different denoising approaches

Figure 9 shows the denoising result of real patient brain PET data. Only NLM, BM3D and GBM4D results were shown for the superior performance shown in the previous section. The boundary of the ventricle can be more clearly shown in the GBM4D denoised image, especially for frames of lower counts. Figure 10 shows the TACs of white matter and gray matter in real patient data. The superior temporal denoising performance of GBM4D can be also observed in real patient data.

## Discussion

This work proposed a new approach of block matching and collaborative filtering method using guide image and 4D filtering designed for dynamic PET images. The guide image combined with coarse prefiltering prevents mismatching in the grouping process, which leads to superior performance in edge-preserving in both computer-simulating images and real patient images. The mismatching during the grouping process can be significantly reduced, which can be observed from the reduction of artifacts in reconstructed images using BM3D and GBM4D denoised sinograms. 4D filtering provided a sparser spectrum of image blocks, leading to the significantly improved temporal and spatial denoising performance of GBM4D compared with the traditional BM3D method. Therefore, TACs of different tissues can be more accurately estimated and the denoising performance of GBM4D when applied to images of lower counts are superior.

For quantitative evaluation, considering the phantom of only physical decay and the phantom of both biomedical decay and physical decay, SSIM and PSNR are both significantly improved using the GBM4D approach. The indexes showed that GBM4D can successively remove the Poisson noise in the dynamic PET sinogram. The RMSE of TACs in ROI in digital brain phantoms was significantly reduced when applying GBM4D. The kinetic analysis of different denoised reconstructed dynamic PET images will be studied in future work.

**Fig. 11:**
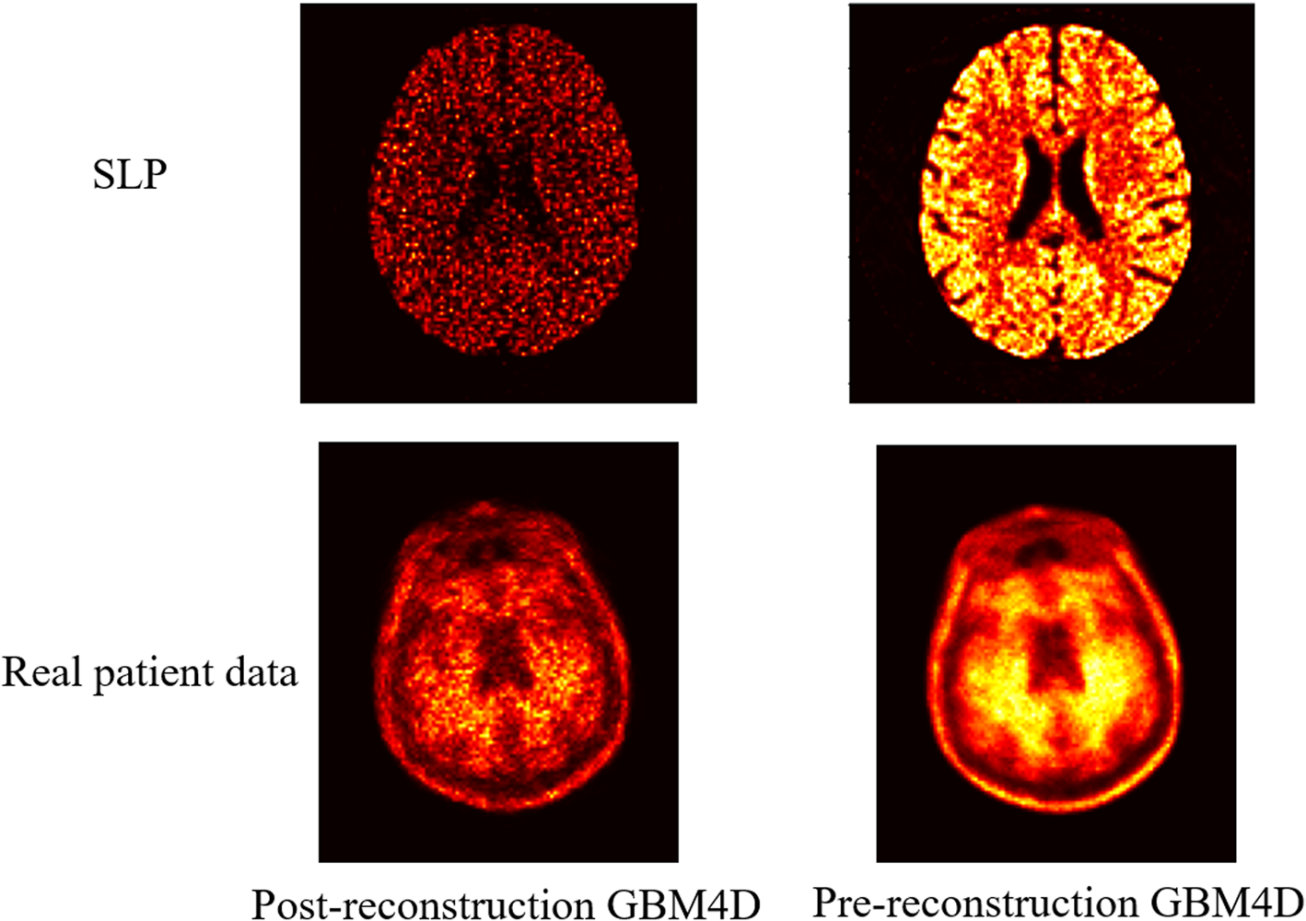
A slice of reconstructed dynamic PET images in the frame of lowest counts for SLP and real patient data after applying post-reconstruction GBM4D and pre-reconstruction GBM4D

Previous research also developed various types of block matching and collaborative approaches [7–10]. When considering dynamic PET sinogram image, BM5D can also be applied to remove the Poisson image. However, the computational burden would be dramatically increased. Using a guided image in GBM4D can reduce the computational burden during the grouping process and prevent mismatching. Most works aimed at denoising images after OSEM reconstruction using block matching and collaborative approaches. In this way, the computational burden can be reduced. The noise of reconstruction images is not exact Poisson noise but has the form of Poisson noise [10]. The Anscombe transformation and its inverse transformation would lead to bias in activity and inferior performance of denoising approaches, as shown in Fig. 11.

GBM4D, when applied to dynamic PET with more frames, has greater potential for image quality improvement compared with BM3D, for more information is contained in the temporal aspect during the collaborative process and relatively fewer chances of mismatching using guide image. Only eight frames were considered in this work to evaluate the performance of such a denoising approach to avoid the dramatic differences between the sparsity of 3D blocks and 4D blocks spectrum, which helps to reduce the possibility of mismatching in BM3D.

A great amount of image-denoising methods based on deep convolution net neural (CNN) have been developed [37–40]. Further work would be done to compare GBM4D with CNN-based approaches. GBM4D, as a new non-local denoising method, suffered from the same disadvantage shared with other non-local methods, which is the computational burden. GBM4D can be transplanted to GPU devices, which can significantly reduce the computation time. Further comparison between BM5D and GBM4D will be conducted in the future when acceptable computational burden for both methods were achievable.

## Conclusion

In this study, we developed a guided block matching and 4-D transform domain filter projection denoising method for dynamic PET image reconstruction and thoroughly investigate the performance of the approach. GBM4D shows great denoising and edge-preserving function. The temporal and spatial denoising performances were both validated qualitatively and quantitatively. Additionally, the use of a guide image in the block-matching process and 4-D filtering allowed for the reduction of artifacts and improved accuracy in TACs estimation. The GBM4D outperformed BM3D in terms of sparsity and denoising performance. Quantitative evaluation using SSIM, PSNR, and RMSE of TACs showed that GBM4D can effectively remove Poisson noise in dynamic PET sinograms. GBM4D, as a new form of block matching and collaborative denoising method, can significantly improve the denoising performance for dynamic PET image reconstruction both spatially and temporally compared with traditional BM3D.

## Data Availability

The data used in this study are available from the corresponding author on reasonable request.
